# Characterization and Performance Evaluation of Cotton Fabrics Functionalized via In Situ Green Synthesis of Silver Nanoparticles Using *Solanum tuberosum* Peel Extract

**DOI:** 10.3390/polym17192598

**Published:** 2025-09-25

**Authors:** Nonsikelelo Sheron Mpofu, Josphat Igadwa Mwasiagi, Cleophas Achisa Mecha, Eric Oyondi Nganyi

**Affiliations:** 1 Department of Apparel Engineering and Textile Processing, Berlin University of Applied Sciences—HTW Berlin, 12459 Berlin, Germany; 2 Department of Manufacturing, Industrial and Textile Engineering, School of Engineering, Moi University, Eldoret P.O. Box 3900-30100, Kenya; igadwa@gmail.com (J.I.M.); ericoyondi@gmail.com (E.O.N.); 3 Department of Environmental Science, University of Arizona, Tucson, AZ 85721, USA; achemeng08@gmail.com; 4 Renewable Energy, Environment, Nanomaterials and Water Research Group, Department of Chemical and Process Engineering, School of Engineering, Moi University, Eldoret P.O. Box 3900-30100, Kenya

**Keywords:** green synthesis, silver nanoparticles, *Solanum tuberosum* extract, potato peels, antibacterial textiles, sustainable nanotechnology, cotton functionalization, nanoparticle characterization

## Abstract

The functionalization of textiles with nanomaterials through green synthesis offers a promising pathway for sustainable material innovation. This study explores the in situ green synthesis of silver nanoparticles (AgNPs) onto cotton fabrics using *Solanum tuberosum* (potato) peel extract as a natural reducing and stabilizing agent. The synthesis conditions were optimized by varying silver nitrate concentration, extract volume, temperature, pH, and reaction time, after which the optimized protocol was applied for fabric treatment. The presence and distribution of AgNPs were confirmed through UV-Visible spectroscopy, Fourier-transform infrared spectroscopy, scanning electron microscopy and dynamic light scattering. The treated fabrics demonstrated strong and durable antibacterial performance, with inhibition zones of 23 ± 0.02 against *Escherichia coli* and 16 ± 0.01 against *Staphylococcus aureus.* Notably, antibacterial activity was retained even after 20 washing cycles, demonstrating the durability of the treatment. Mechanical testing revealed a 32.25% increase in tensile strength and a corresponding 10.47% reduction in elongation at break compared to untreated fabrics, suggesting improved durability with moderate stiffness. Air permeability decreased by 8.8%, correlating with the rougher surface morphology observed in Scanning Electron Microscopy images. Thermal analysis showed a decrease in thermal stability relative to untreated cotton, highlighting the influence of AgNPs on degradation behavior. Overall, this work demonstrates that potato peel waste, an abundant and underutilized biomass, can be used as a sustainable source for the green synthesis of AgNP-functionalized textiles. The approach provides a cost-effective and environmentally friendly strategy for developing multifunctional fabrics, while supporting circular economy goals in textile engineering.

## 1. Introduction

Textile materials are fundamentally composed of polymers, that is, long-chain macromolecules that can be natural or synthetic in origin. These polymers provide the structural backbone and functional properties of textile fibers. Natural fibers such as cotton are primarily composed of cellulose, a polymer consisting of long chains of β-glucose joined together by 1,4 glycosidic bonds. The abundant hydroxyl groups in cellulose not only contribute to cotton’s hydrophilicity and comfort but also make it a fertile medium for microbial colonization, especially under moist and warm conditions. This microbial susceptibility can lead to undesirable effects such as odor development, fabric degradation and health-related issues, particularly in medical and personal care textiles [1,2]. To counter microbial contamination, a variety of nanomaterials have been explored and integrated into textile substrates. Unlike other nanoparticles, such as titanium dioxide (TiO_2_) [3], copper oxide (CuO) [4], which have been explored for antimicrobial purposes, silver nanoparticles remain the most widely used and commercially relevant antimicrobial nanomaterials for textiles. Their nanoscale size allows for effective interaction with fibers and microbial cells, while silver’s superior thermal and electrical conductivity, low contact resistance, and multiple oxidation states enable its broad-spectrum antimicrobial efficacy [5]. The antimicrobial efficacy of silver is attributed to its ability to deactivate intracellular proteins, thereby inhibiting the growth of microorganisms, Ref. [6] making it an invaluable component in medical and industrial applications. The effectiveness of silver nanoparticles against Gram-negative and Gram-positive bacterial pathogens including *S. aureus*, *Klebsiella pneumoniae*, *E. coli*, *Pseudomonas aeruginosa*, methicillin-resistant *Staphylococcus aureus* and methicillin-sensitive *Staphylococcus aureus* has been demonstrated [7,8].

In textile applications, AgNPs are valued not only for their potential antimicrobial activity, but also for their ability to impart durable functionality without compromising comfort, breathability or mechanical strength. Their strong binding to cellulose fibers enhances wash durability, making them particularly suited for medical textiles, sportswear and personal care products [9,10]. Recent advancements in the field of nanotechnology have focused on the eco-friendly synthesis of AgNPs and their application to cotton fabrics for antimicrobial properties. These AgNPs can be applied via the chemical reduction of silver nitrate (AgNO_3_) using both chemical and biological routes [11]. Researchers have used various eco-friendly reducing agents, including sugar [12], *Solanum tuberosum* peel extract [13], fungi isolated from medicinal plants [14], banana peels [15], garlic cloves [16], lemon peel and tea extracts [17,18,19], as well as band starch residue on cotton fabrics [20]. The use of plant-based materials for nanoparticle synthesis offers significant advantages due to the diverse functional groups present in plants, such as phenols, ketones, amines, hydroxyl groups, carbonyl groups, and flavonoids, which facilitate the reduction process. Various plant components, including stem bark, seeds, leaves, fruits, flowers, and roots, can be employed in nanoparticle synthesis. Among these, potato peels are particularly promising; with an annual production of approximately 140,000 tons of waste, they are an abundant and sustainable source of bioactive compounds, including phenolic acids and flavonoids [21,22,23]. These compounds serve a dual function in the green synthesis of silver nanoparticles, acting as both reducing and capping agents to facilitate nanoparticle formation while stabilizing them against aggregation and oxidation [24]. Beyond their role in nanoparticle synthesis, the bioactive compounds impart antimicrobial, antioxidant and antifungal properties, thereby combining sustainable waste valorization with the development of functional, high-performance textiles.

The application of synthesized nanoparticles onto textile substrates is commonly achieved through two main approaches, known as ex situ and in situ techniques. The in situ synthesis of AgNPs onto textile fabrics is emerging as a preferred approach due to its improved antimicrobial properties, durability and sustainability. The process uses the bioactive compounds present in plant extracts that facilitate nanoparticle formation while reducing the use of synthetic chemicals. Recent studies have compared in situ and ex situ techniques for embedding Ag-TiO_2_ nanoparticles into polyester/cellulosic fabrics. In situ synthesis yields smaller, more uniformly distributed nanoparticles with higher surface concentration and superior wash durability [25]. It also enhances nanoparticle stabilization efficiency and reduces agglomeration, improving functional performance [26,27,28,29]. Key factors influencing synthesis efficiency include fabric surface properties, chemical composition and reaction conditions such as pH, temperature and synthesis time [30]. Controlling synthesis conditions enables precise control of nanoparticle size and distribution, which directly impacts antimicrobial effectiveness and fabric performance. Tailoring these parameters based on fabric characteristics and desired properties enables the development of functionalized textiles with enhanced stability and longevity.

This study explores the green in situ synthesis of silver nanoparticles onto cotton fabrics using *Solanum tuberosum* peel extract as a natural reducing and stabilizing agent. The characterization of nanoparticle-treated fabrics was performed to determine successful nanoparticle synthesis and assess their interaction with the textile matrix. Functional properties such as antimicrobial activity, durability and surface morphology were also evaluated. Despite growing interest in green synthesis approaches, many studies focus primarily on the synthesis and antimicrobial efficacy of nanoparticles in solution, with limited investigation into how these particles behave once applied to natural fiber substrates such as cotton. Moreover, there is a limited systematic evaluation of the performance characteristics, such as wash durability, air permeability and thermal properties of in situ green-synthesized silver nanoparticles on cotton fabrics. The work aims to address this gap by examining the physicochemical properties of the AgNP-treated fabrics, including surface morphology, elemental composition, and structural changes. Additionally, the study evaluates the fabric’s durability and antibacterial functionality to assess the effectiveness of the in situ synthesized AgNPs.

By using agricultural waste as a bioresource, this approach aligns with circular economy principles by repurposing the waste for functionalizing textiles in an environmentally friendly manner. It provides a sustainable alternative to conventional antimicrobial treatments, reducing reliance on chemical-based synthesis while mitigating associated health and environmental risks. The findings contribute to advancements in bio-based nanotechnology and functional textiles, offering insights into environmentally friendly approaches for enhancing polymer-based materials.

## 2. Materials and Methods

### 2.1. Materials

The peels used were from *Shangi* potatoes collected from a farm in Uasin Gishu, Kenya. Plain woven, mercerized, bleached and scoured cotton fabric was purchased from Rivatex East Africa Limited in Eldoret, Kenya and had the following characteristics: 128 g/m^2^, 34 picks/inch, 72 ends/inch, 28 tex warp count and a thickness of 0.002 mm. AgNO_3_ was purchased from Science Lab Limited, Nairobi, Kenya. Bacteria strains and reagents for antibacterial assays were purchased from Sigma-Aldrich (St Louis, MO, USA) and ThermoFisher Scientific (Walham, MA, USA). All reagents and chemicals used in the study were of analytical grade and were used without further purification.

### 2.2. Methods

#### 2.2.1. Green Synthesis of AgNPs

The green synthesis of AgNPs was conducted using *Solanum tuberosum* peel extract as a reducing agent, based on a previously described method [31]. In this process, 300 µL of a 1 mM AgNO_3_ solution was heated to 100 °C in an Eppendorf tube. Thereafter, 100 µL of potato peel extract (PPE) was added to reach a total volume of 400 µL. The mixture was shaken in the dark at 500 rpm for one hour. A visible change from colorless to yellowish-brown indicated silver nanoparticle formation, attributed to the surface plasmon resonance (SPR) of AgNPs. This transformation is facilitated by the phytochemicals in the extract which act as natural reducing and stabilizing agents. Since the phytochemical composition varies across plant species, optimization of synthesis conditions is crucial for each plant-based extract.

Key synthesis parameters including extract concentration, AgNO_3_ to extract ratio, pH, temperature, stirring rate and reaction time were systematically investigated to determine the optimal conditions for nanoparticle formation. The presence and characteristics of silver nanoparticles were confirmed using the POLARstar Omega microplate reader software version 5.11 R3 (BMG-Labtech, Ortenberg, Germany) UV-visible spectroscopy by identifying SPR peaks within the range of 400–500 nm [32,33]. A higher intensity and narrower SPR peak were interpreted as indicating a higher concentration of well-dispersed nanoparticles with lower polydispersity.

To study the effect of extract concentrations, serial dilutions of PPE (100%, 50%, 25%, 12.5% and 6.25%) were prepared and reacted with AgNO_3_ at 100 °C and 500 rpm for one hour. The concentration that yielded the most favorable SPR characteristics was selected for subsequent experiments. Similarly, various AgNO_3_ to extract volume ratios were tested, and the ratio yielding the most intense and narrow SPR peak was used going forward.

The initial measurement of the potato peel extract’s pH was 5.8. To create the alkaline environment required for efficient nanoparticle synthesis, the pH was systematically adjusted to the range of 6 to 12 using 1 M NaOH. The effect of temperature was also evaluated by conducting reactions at 25 °C, 50 °C, 70 °C and 100 °C. For each experimental condition, UV-Vis spectroscopy was used to monitor the SPR, to determine the optimal pH and temperature for nanoparticle formation.

The influence of stirring speed was evaluated at 0, 300, 500 and 700 rpm. Stirring affects particle size and agglomeration, with either too slow or too rapid mixing potentially leading to larger, less stable nanoparticles. The optimal mixing speed was identified from UV-Vis spectral data. Finally, the effect of reaction time was assessed by recording absorption spectra at intervals from 10 min to 4 h. The combination of optimal parameters; extract concentration, AgNO_3_ ratio, pH, temperature, stirring speed and synthesis time was determined and used for subsequent experiments.

Nanoparticles were synthesized at determined conditions and then characterized using the UV-Vis spectroscopy, DLS and SEM. UV-Vis spectroscopy was performed using the POLAstar Omega microplate reader and confirmed nanoparticle formation through the detection of SPR peaks in the 400–500 nm range, within a scanning range of 300–800 nm. DLS analysis was performed using the Zetasizer Nano ZS90 (Malvern Panalytical, Malvern, UK) and it assessed the particle size distribution and zeta potential. For particle sizing, 1 mL of the nanoparticle suspension was equilibrated for 2 min in a cuvette, with the hydrodynamic diameter averaged over five runs. Zeta potential was measured by injecting the suspension into a zeta cell, with all readings taken in triplicate. Morphological characterization was performed using a Zeiss high-resolution SEM. Powdered nanoparticles were mounted on a carbon strip and placed in a vacuum chamber, and images were captured at 5.0 kV to analyze shape and surface features of the green-synthesized nanoparticles.

The optimum parameters were also used in the in situ synthesis of AgNPs onto cotton fabrics.

#### 2.2.2. In Situ Green Synthesis of Silver Nanoparticles onto Cotton Fabric

**Figure 1:**
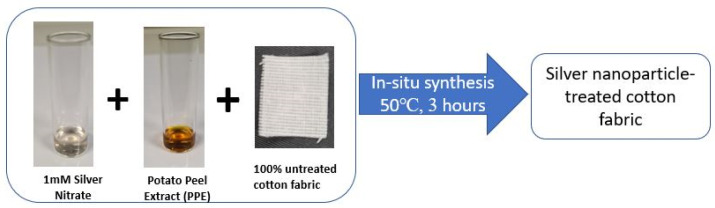
Direct in situ synthesis of nanoparticles onto cotton fabric.

The deposition of silver nanoparticles onto cotton fabric was achieved through a one-pot in situ synthesis approach, using 1 mM AgNO_3_ solution as the metal precursor and potato peel extract as the natural reducing agent (Figure 1). This method was adapted from previously reported procedures [14,34,35] and applied under optimized synthesis conditions.

Prior to treatment, bleached, scoured and mercerized 100% cotton fabrics were washed using a non-ionic detergent at 60 °C for 40 min. The washed fabrics were cut into pieces weighing 300 mg and placed into individual tubes containing 333 µL of AgNO_3_ solution, ensuring that each fabric piece was completely immersed in the solution. The tubes were heated to 50 °C on a magnetic stirrer, after which 67 µL of potato peel extract prepared at a 25% concentration and adjusted to pH 12 was added. The reaction mixtures were stirred continuously at 500 rpm for 3 h. Upon completion, the fabrics were thoroughly rinsed to eliminate residual reagents. A visible color change from white to golden brown confirmed the formation of silver nanoparticles on the cotton surface. Finally, the treated fabrics were air-dried at room temperature for 24 h in a drying cabinet before undergoing characterization and antimicrobial testing.

For comparison, control fabrics were prepared by treating them with potato peel extract alone, allowing assessment of the contribution of the potato peel extract independently. The reaction conditions and reagent-to-fabric ratios were designed to facilitate potential scale-up, where both fabric mass and reagent volumes can be proportionally increased while maintaining the same temperature, stirring speed and reaction time.

#### 2.2.3. Durability of the Treated Fabrics to Washing

Fabric samples were washed following the ISO C10:2006 standard [36] for wash fastness. Washing was carried out at 70 °C for 45 min using 100 steel balls, simulating five regular wash cycles. This was followed by a 10 min hot rinse at 40 °C in plain water. The fabrics were then air-dried overnight under controlled laboratory conditions. The washing process was repeated to simulate 10 and 20 wash cycles. Qualitative antibacterial tests against *E. coli* and *S. aureus* were conducted on both washed and unwashed treated fabrics.

#### 2.2.4. Qualitative Efficacy Testing of the Treated Fabrics After Bacteria

The antibacterial activity of the treated fabrics was evaluated using the agar disk diffusion method as described in the literature [37]. In brief, overnight-cultured bacterial suspensions were uniformly spread onto nutrient agar plates. The treated and untreated fabric samples were cut into 6 mm disks and carefully placed in the inoculated agar plates. The plates were incubated at 37 °C for 16 to 18 h. After incubation, the diameter of the clear zone surrounding each fabric disk, known as the zone of inhibition, was measured in millimeters to assess antibacterial efficacy. Antibiotic-impregnated disks were used as positive controls, while untreated cotton fabrics were used as negative controls. Additionally, fabrics treated with potato peel extracts only were used for comparison. All tests were performed in triplicate for each bacterial strain to ensure accuracy and reproducibility.

#### 2.2.5. Characterization of the Physical and Chemical Properties of the Treated Samples

The physical and chemical characteristics of the fabric samples were evaluated using various analytical techniques. To study the surface morphology, a Zeiss high-resolution scanning electron microscope fitted with energy dispersive spectroscopy was used. Fabric samples, both treated and untreated, were cut into 5 mm by 5 mm pieces, mounted on a conductive carbon strip, and sputter-coated with gold [37]. The deposition of silver nanoparticles on the fabric surface was observed through the resulting SEM images.

Chemical bonding in the untreated and silver nanoparticle-treated cotton fabrics was examined using a Jasco FT/IR-6600 Type A spectrometer (Jasco Corporation, Tokyo, Japan), which includes a standard light source and triglycine sulfate (TGS) detector. A potassium bromide (KBr) pellet was prepared by mixing ground fabric pieces with KBr, and the spectra were recorded with a resolution of 4 cm^−1^ across a wavelength range of 400 to 4000 cm^−1^.

Thermal stability was assessed using a Simultaneous TGA/DSC analyzer STA 6000 (PerkinElmer, Shelton, CT, USA) under air atmosphere, with heating at a rate of 10 °C per minute from room temperature to 800 °C. Approximately 10 mg of the finely cut fabric sample was placed in an aluminum oxide pan for analysis.

The air permeability of the fabric was measured according to ISO 9237 [38] using an Air Permeability Tester under standard atmospheric conditions. Measurements were performed at a constant air pressure over a 20 cm^2^ area. Five samples from each fabric type were tested, and the average airflow rate, expressed in mm/second, was calculated.

Tensile strength was tested using a Testometric Micro 500 universal tensile tester (Rycobel, Deerlijk, Belgium), following ISO 13934:2013 [39] guidelines for determining the tensile properties of fabrics. The fabric samples were preconditioned under standard atmospheric conditions and cut into rectangular strips. Each strip was clamped with the longer side aligned to the direction of force. Testing was carried out at a speed of 100 mm/min until the fabric broke, and the maximum force at breakage was recorded. Five samples were tested and the average value reported.

#### 2.2.6. Statistical Analysis

All experimental results were presented as mean plus or minus the standard deviation (mean ± SD) of three independent replicates. Statistical analysis was performed using a one-way analysis of variance (ANOVA) to determine significant differences among treatments. Post hoc comparisons of the means were carried out using Fisher’s least significant difference (LSD) test within Design Expert version 13 software, with significance set at *p* < 0.05. The number of replicates for each treatment group and bacterial strain was explicitly three, ensuring reproducibility and statistical validity.

## 3. Results and Discussion

### 3.1. Green Synthesis of Silver Nanoparticles

#### 3.1.1. Visual Assessment of Green-Synthesized Nanoparticles

**Figure 2:**
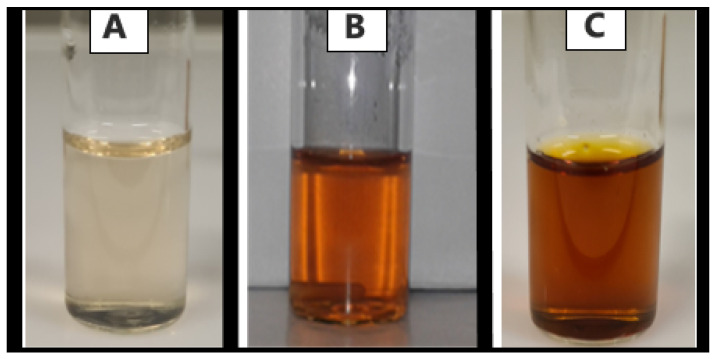
Visual color-change in synthesized nanoparticles at (**A**) 0 min, (**B**) 30 min and (**C**) 3 h.

Silver nanoparticles exhibit a strong absorption band and display a characteristic color in solution due to surface plasmon resonance [32]. This color change from a pale brown (Figure 2A) to a yellowish-brown hue (Figure 2B) indicates nanoparticle formation [40]. Initially, when the potato peel extract was added to the AgNO_3_ solution, the mixture appeared very light and clear brown. After 30 min, the color turned golden-brown and continued to deepen into a darker brown after 3 h of reaction (Figure 2C).

Further confirmation of silver nanoparticle synthesis was obtained through UV-Vis spectroscopy within the 300–800 nm range. Typically, silver nanoparticles display a surface plasmon resonance peak between 400 and 500 nm [32,41]. A shift to lower wavelengths suggests the formation of smaller nanoparticles, while a shift to higher wavelengths indicates larger nanoparticles [42].

#### 3.1.2. Determination of Suitable Green-Synthesis Parameters

**Figure 3:**
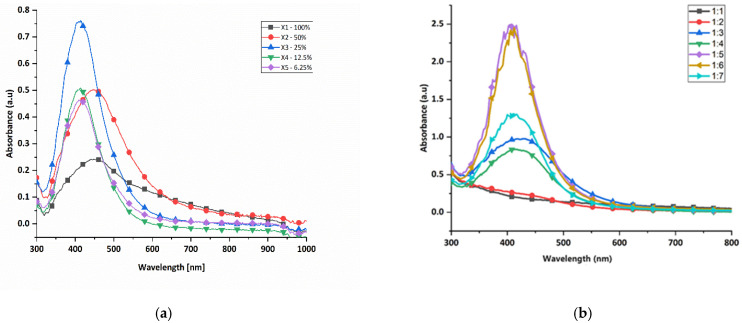
UV-vis absorption spectra of silver nanoparticles synthesized at (**a**) different concentrations of potato peel extract and (**b**) different extract to AgNO_3_ volume ratios.

To evaluate the optimal conditions for the green synthesis of silver nanoparticles, tests were first conducted to assess the effect of the potato peel extract concentration. Thereafter, tests were performed to determine the effect of the extract-to-AgNO_3_ volume ratio. The corresponding spectra for both parameters are presented in Figure 3.

As the potato peel extract concentration increased from 6.25% to 25%, there was a rise in absorbance and a narrower SPR peak (Figure 3a), indicating the formation of more nanoparticles with a uniform size distribution [43]. Higher concentrations increased the phytochemical content, enhancing the reduction rate of AgNO_3_ to AgNPs [19,44]. However, at 50% extract concentration, the SPR peak broadened, suggesting polydispersity due to rapid nanoparticles formation, aggregation and the formation of bulk silver [45]. The optimal concentration for green synthesis was determined to be 25%.

The effect of potato peel extract to AgNO_3_ volume ratio was examined using different ratios from 1:1 to 1:7 (Figure 3b). At lower ratios of 1:1 and 1:2, little to no nanoparticle formation occurred, as indicated by broader SPR peaks and lower absorbance [46]. As the ratio increased to 1:5, the absorbance and SPR peak intensity increased, showing more uniform nanoparticles [47]. At ratios higher than 1:5, the synthesis decreased and the SPR peak broadened, indicating polydispersity. It is likely that as the volume ratio of the AgNO_3_ increases, there is insufficient potato peel extract for the reaction with silver ions, hence the reduced synthesis [48]. The optimal ratio for nanoparticle synthesis was 1:5, which was used in subsequent experiments.

**Figure 4:**
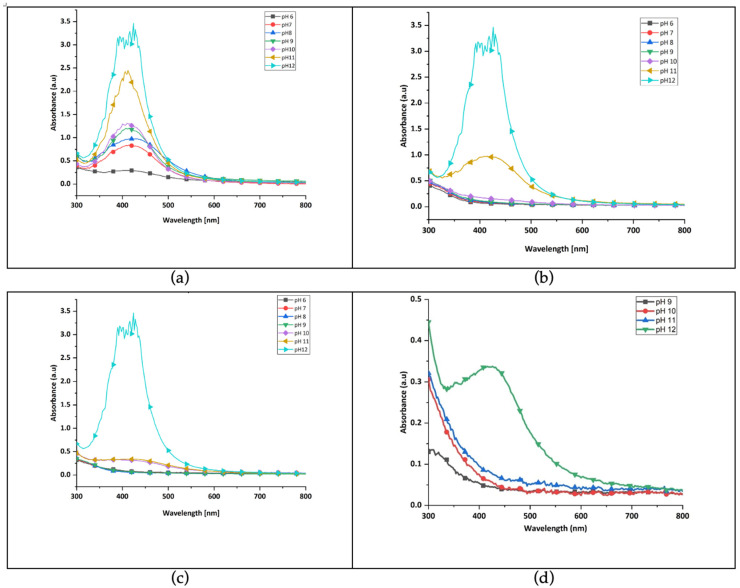
Combined effect of pH and temperature at (**a**) 99 °C, (**b**) 70 °C, (**c**) 50 °C, and (**d**) 25 °C.

The combined influence of pH and temperature on green synthesis of silver nanoparticles revealed that higher pH levels, that is, alkaline levels resulted in greater nanoparticle formation. This supports previous findings that alkaline conditions enhance green synthesis [33,45]. Although synthesis was successful across all pH levels at 99 °C (Figure 4a), lower temperatures were investigated to promote energy efficient and cost-effective processes [49]. At 70 °C (Figure 4b), 50 °C (Figure 4c) and 25 °C (Figure 4d), no significant SPR peaks appeared for pH 6–10. However, distinct peaks were observed at pH 11 and 12, with the highest intensity at 50 °C and pH 12. These conditions were identified as optimal for further experiments.

**Figure 5:**
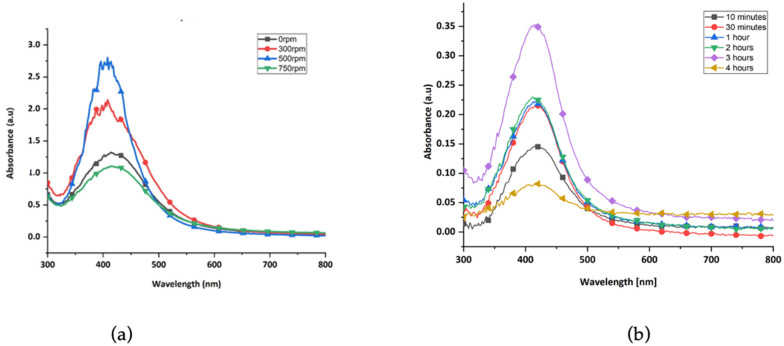
(**a**) UV-vis absorption spectra of silver nanoparticles synthesized by varying the stirring speeds. (**b**) UV-vis spectra of silver nanoparticles synthesized at different reaction times.

The UV-Vis spectra for the effect of stirring speed on green synthesis of nanoparticles are shown in Figure 5a. As the speed increased from 0 to 300 rpm and then to 500 rpm, absorbance also increased, indicating enhanced nanoparticle formation. However, at speeds above 500 rpm, the absorbance decreased, and the peak broadened, suggesting the formation of larger, polydisperse nanoparticles due to excessive aggregation. Low stirring speeds led to reduced reaction rates. Therefore, 500 rpm was identified as the optimal stirring speed and used in further experiments.

The synthesis time also influenced nanoparticle formation, and the UV-vis spectra are shown in Figure 5b. Silver nanoparticles began forming within 10 min, with absorbance increasing and peaks becoming sharper over time. The most significant nanoparticle formation occurred at 3 h, evidenced by the highest absorbance and sharpest SPR peak. At 4 h, the peak became broader, and the absorbance dropped, indicating aggregation and formation of larger particles. Thus, synthesis time of 3 h was considered optimal.

The synthesis yielded the most favorable results when a 25% concentration of potato peel extract was used in combination with a 1:5 extract-to- AgNO_3_ volume ratio. The silver nanoparticles with the highest absorbance and the most distinct surface SPR peak were obtained at a temperature of 50 °C and a pH of 12, with continuous stirring at 500 rpm and at a reaction time of 3 h. These conditions were adopted for all subsequent experiments.

#### 3.1.3. Characterization of Nanoparticles Synthesized Using Optimum Values

**Figure 6:**
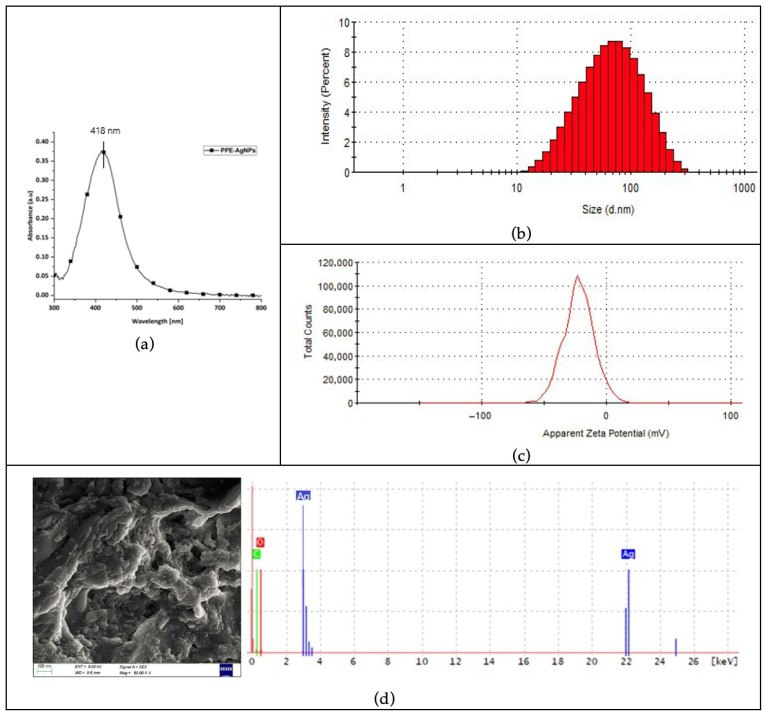
(**a**) UV-vis spectrum of silver nanoparticles green-synthesized with potato peel extract, (**b**) Size distribution of the green-synthesized silver nanoparticles, (**c**) Zeta potential analysis of the green-synthesized silver nanoparticles, (**d**) SEM image and EDX spectra of green synthesized silver nanoparticles (Red: Oxygen, Green: Carbon and Blue: Silver).

The UV-vis spectrum of the nanoparticles synthesized with the optimum parameters is shown in Figure 6a. The single peak between 400 nm and 500 nm is indicative of the SPR of silver nanoparticles [32,33]. This peak not only confirms nanoparticle formation but also provides insights into particle size and uniformity, as the SPR wavelength shifts slightly with particle size and aggregation state. The narrowness of the peak suggests relatively uniform particle size with minimal aggregation.

Dynamic light scattering (DLS) was used to determine the hydrodynamic diameter and size distribution (Figure 6b) of the green-synthesized nanoparticles. The average hydrodynamic diameter of 50.18 nm falls within the typical DLS-measured nanoparticle range of 20–100 nm, confirming successful synthesis [45,50,51]. The polydispersity index (PDI) of 0.282 indicates moderate polydispersity and suggests that the synthesis method produced nanoparticles with reasonably consistent sizes [52].

Zeta potential analysis (Figure 6c) revealed an average value of −33 mV, indicating moderate colloidal stability. The negative charge potential facilitates electrostatic repulsion between nanoparticles, preventing aggregation and promoting dispersion in solution [53,54]. These results confirm that the nanoparticles were stable, well dispersed and suitable for further applications. This stability is essential for downstream applications, such as coating textiles, where uniform particle distribution enhances functional performance.

The SEM results in Figure 6d revealed that the green-synthesized silver nanoparticles were predominantly spherical, with some larger clusters likely formed due to partial agglomeration. The surface morphology confirmed typical characteristics of nanoparticles produced via green synthesis, which typically yield spherical or quasi-spherical nanoparticles [45,48,49]. Energy-dispersive X-ray analysis showed a strong silver peak at 3 keV, confirming the presence of silver nanocrystals [49,55]. The absence of nitrogen indicated the complete reduction of AgNO_3_. Peaks for carbon and oxygen were also present, likely originating from the organic compounds in the potato peel extract used in the synthesis, which acted as both reducing and capping agents, stabilizing the silver nanoparticles during formation.

### 3.2. Visible Effects of the Silver Nanoparticles on Cotton Fabric

**Figure 7:**
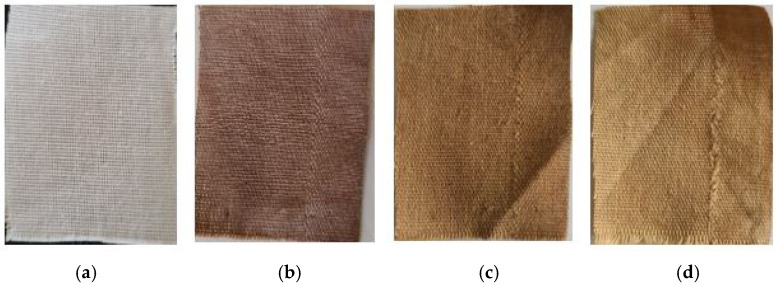
100% cotton fabrics. (**a**) untreated, (**b**) treated with AgNPs before washing, (**c**) treated with AgNPs after 10 washes, (**d**) treated with AgNPs after 20 washes.

The visual changes observed on the cotton fabric following in situ synthesis of silver nanoparticles are presented in Figure 7. The fabric shifted in color from white to brown, indicating the successful formation of silver nanoparticles directly on the fabric surface through the reduction of Ag^+^ to Ag^0^ [40]. The use of potato peel extract as both a reducing and capping agent played a key role in stabilizing the nanoparticles on the fiber surface, preventing rapid aggregation and oxidation during synthesis. The bioactive compounds in the extract, such as phenolic acids and flavonoids, adsorb onto the nanoparticle surface, forming a protective layer that enhances their adhesion to the cotton fibers. After 10 wash cycles, the color intensity diminished, and it faded even more noticeably after 20 washes, suggesting a gradual loss of silver nanoparticles due to washing [37].

### 3.3. Antibacterial Efficacy Testing of the Treated Fabric Samples

**Figure 8:**
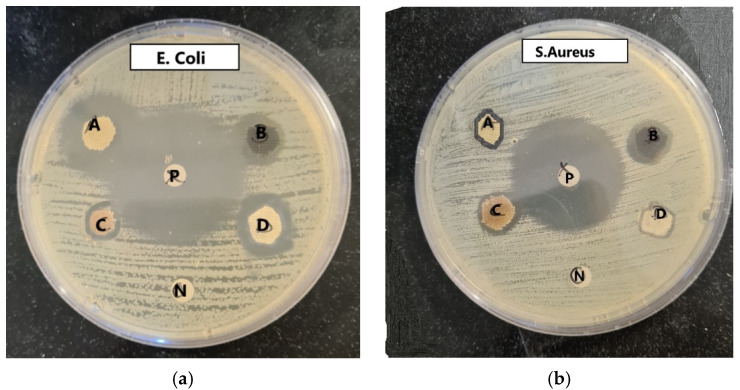
Antibacterial efficacy of different fabrics against (**a**) *E. coli* and (**b**) *S. Aureus*. A—PPE treated fabrics before washing, B—AgNPs treated fabric before washing, C—AgNPs treated fabric after 20 washes, D—PPE treated fabric after 20 washes, P—Ampicillin disk (positive control), N—untreated fabrics (negative control).

The antibacterial properties of cotton fabrics treated with silver nanoparticles synthesized from potato peel extract (PPE-AgNPs) were evaluated using the fabric disk diffusion method against two bacterial strains: Gram-positive *S. aureus* and Gram-negative *E. coli*. Untreated cotton fabric served as the negative control, while fabric treated with the antibiotic ampicillin acted as the positive control. Additionally, fabrics treated solely with potato peel extract were tested to assess the combined antimicrobial effect of the extract and the synthesized silver nanoparticles. Figure 8 presents the results of this analysis. The zones of inhibition are shown in Table 1.

Clear zones of inhibition around the treated fabric disks confirmed the presence on antibacterial activity. No inhibition zone was observed around the untreated control, indicating a lack of antimicrobial effect. Notably, the AgNP-treated fabrics exhibited larger inhibition zones compared to the PPE-treated samples, demonstrating stronger antibacterial performance and highlighting the enhanced antimicrobial efficacy achieved through green synthesis of silver nanoparticles using potato peel extract. However, a decline in antibacterial activity was observed in both treated samples following 20 washing cycles. The findings align with previous studies reporting that fabrics coated with nanoparticles display antibacterial activity against *E. coli* and *S. aureus*, which diminishes with laundering [26,56,57].

### 3.4. Characteristics of the Fabric Treated with Silver Nanoparticles

#### 3.4.1. Morphology and Elemental Analysis

**Figure 9:**
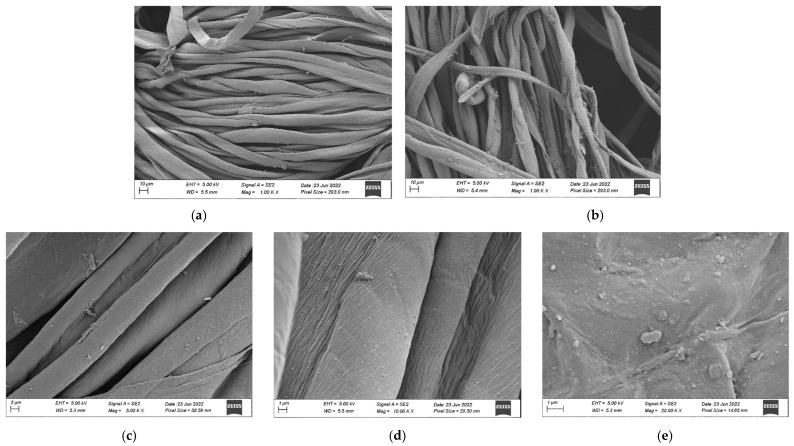
SEM images of cotton fabrics. (**a**) untreated cotton fabric at 1 kX magnification, (**b**) treated fabric at 1 kX magnification, (**c**) treated fabric at 5 kX magnification, (**d**) treated fabric at 10 kX magnification, (**e**) treated fabric at 20 kX magnification.

The surface morphology of cotton fabrics following the in situ synthesis of silver nanoparticles was examined using SEM. As shown in Figure 9, the untreated control sample (Figure 9a) displayed smooth, longitudinally aligned fibrils characteristic of native cotton fiber, without any surface particles. In contrast, the cotton fabrics treated with silver nanoparticles (Figure 9b–e) appeared rougher due to the presence of a thin nanoparticle layer coating the fibers. The SEM images showed that the silver nanoparticles were predominantly spherical and were distributed across the fiber surfaces. Some nanoparticles formed clusters, resulting in a mixture of smaller particles and larger agglomerate, an observation consistent with findings from previous studies [58,59,60]. These morphological changes confirmed the successful deposition of silver nanoparticles onto the cotton fabric.

**Figure 10:**
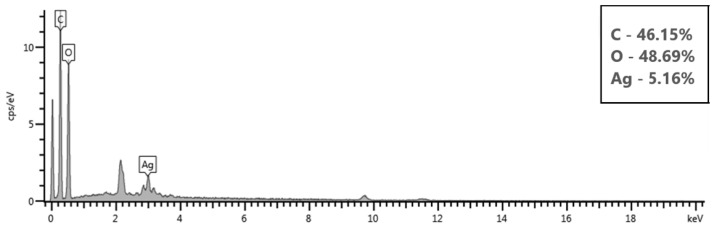
Elemental analysis of the AgNP treated cotton fabric.

The elemental analysis of the cotton fabric treated with silver nanoparticles, shown in Figure 10, displayed a distinct peak at 3 keV, which is a typical signature of silver nanoparticles due to their surface plasmon resonance [61], thereby confirming their presence. Energy Dispersive X-ray (EDX) analysis further indicated that the fabric mainly consisted of carbon (46.15%) and oxygen (48.69%), both of which are key elements of cellulose, the primary component of cotton fibers. Silver was detected in a small amount (5.16%), verifying the successful incorporation of silver nanoparticles onto the fabric. Similar findings were reported in another study, where elemental analysis showed 2.2% silver on the treated cotton, with carbon and oxygen together making up over 90% of the sample’s composition [61]. The presence of silver in relatively small but measurable amounts indicates a thin, uniform nanoparticle coating.

#### 3.4.2. Functional Group Analysis

**Figure 11:**
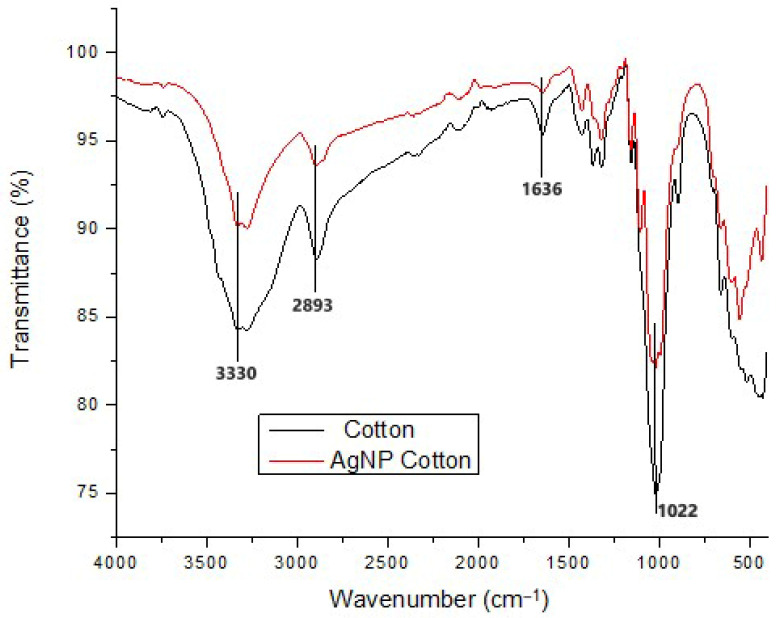
FTIR spectra of untreated cotton fabric and AgNP-treated cotton fabric.

The functional group analysis of cotton fabric before and after treatment with silver nanoparticles reveals no significant changes in the characteristic peaks of cellulose (Figure 11). Both the treated and untreated samples display prominent absorption bands at 3330 cm^−1^ (O–H stretching due to hydrogen bonding), 2893 cm^−1^ (C–H stretching), 1636 cm^−1^ (C=C stretching), and 1022 cm^−1^ (C–O–C stretching). These results indicate that the green-synthesized silver nanoparticles do not alter the chemical structure of the cellulose fibers. Instead, their interaction with the fabric is attributed primarily to physical adsorption and entrapment on the fiber surface.

The reduction and stabilization of silver nanoparticles are facilitated by biomolecules present in the potato peel extract, which was used as the reducing and capping medium. Potato peels are rich in starch, polyphenols, flavonoids, proteins and organic acids. The hydroxyl (-OH) groups from starch and polyphenols typically appear as broad absorption bands around 3300 cm^−1^, while carbonyl groups from flavonoids and organic acids give rise to stretching bands appearing between 3200 and 3400 cm^−1^ and amide bands around 1540 cm^−1^. These groups act as electron donors to reduce Ag^+^ ions to metallic Ag^0^. Once formed, the nanoparticles are surrounded by the same biomolecules, which act as capping agents that prevent aggregation and promote stable attachment to the cotton surface through hydrogen bonding and electrostatic interactions.

The findings agree with those reported in a previous study where AgNPs were synthesized in situ on cotton fabric using ascorbic acid as the reducing agent [62]. The resulting FTIR spectra showed no peak differences between treated and untreated samples, confirming that the cellulose structure remained chemically intact. Similarly, no new peaks were observed when sodium alginate-mediated silver nanoparticles were applied to cotton fabric [58]. However, increased intensities in the peaks associated with C–H stretching, O–H stretching, and C–O stretching, were observed, likely due to the presence of sodium alginate. Silver nanoparticles typically interact with cellulose fibers through physisorption, that is, non-covalent interactions such as Van der Waals forces or electrostatic attractions [63]. As highlighted in an earlier study, this physical adherence does not involve strong chemical bonding and therefore preserves the chemical structure of the cotton fabric [28]. Minor increases in peak intensity, as observed in some studies using capping agents, may reflect additional hydrogen bonding or surface interactions without forming new covalent bonds [60,64].

#### 3.4.3. Thermogravimetric Analysis

**Figure 12:**
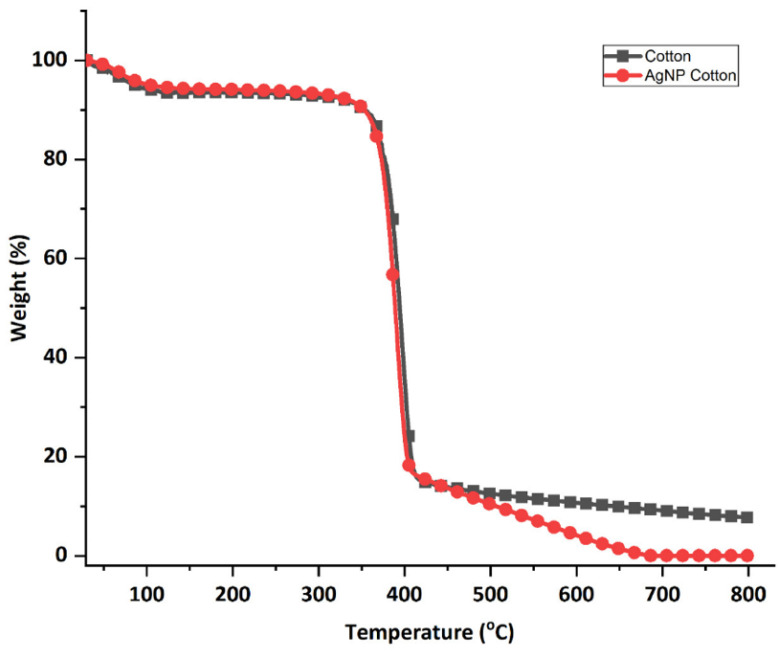
TGA thermograms of untreated cotton fabric and fabric treated with silver nanoparticles (AgNP Cotton).

The thermogravimetric properties of the cotton fabric before and after adding the silver nanoparticles is shown in Figure 12. At the initial heating stage, from room temperature up to 100 °C, both untreated and treated cotton fabrics showed similar weight loss patterns. This slight weight reduction of about 5% continued steadily up to around 350 °C and is attributed to the evaporation of moisture from the fabric. A significant drop in weight occurred between 350 °C and 400 °C for the AgNP-treated fabric, and between 350 °C and 410 °C for the untreated fabric, corresponding to the thermal decomposition of the cotton material [64].

The degradation process continued up to 700 °C; however, the weight loss of the sample treated with silver nanoparticles is higher than the untreated sample. This is due to interactions between the green-synthesized silver nanoparticles and the cellulose in the fabric, which may influence both the rate and mechanism of thermal decomposition [65].

The impact of silver nanoparticles on cotton’s thermal stability remains inconclusive in the literature. Some studies report enhanced thermal stability due to a protective barrier formed by the nanoparticles, which slows down degradation and increases char residue [66]. Conversely, others suggest that silver nanoparticles may lower thermal stability by accelerating cellulose breakdown and reducing the activation energy required for degradation [67]. These contradictory findings highlight that the factors such as nanoparticle size, shape, concentration, distribution, deposition method and fabric type can influence thermal behavior. Moreover, the thermal resistance of AgNP-treated cotton also depends on the testing atmosphere, with nitrogen favoring pyrolytic decomposition while air accelerated oxidative degradation, often amplifying the catalytic role of AgNPs in lowering char yield [67]. This may partly explain the discrepancies across studies. This study used air for analysis and observed reduced thermal stability and a corresponding decrease in char residue in the AgNP-treated cotton fabric.

#### 3.4.4. Air Permeability

The application of silver nanoparticles on cotton fabric resulted in a decrease in air permeability. The untreated fabric exhibited an average air permeability of approximately 6780.43 mm/s, whereas the treated fabric showed a slightly lower value of about 6181.97 mm/s. The observed decrease in air permeability can be directly correlated with the SEM results (Figure 9b–e), which revealed that the nanoparticles formed a thin coating on the fiber surfaces and, in some areas, clustered into small aggregates. This deposition partially blocks the inter-fiber spaces, effectively reducing pore size within the fabric structure. Since air permeability is directly related to the size of pores within the fabric structure [68,69], a reduction in pore size naturally results in lower air permeability. Furthermore, FTIR analysis (Figure 11) confirmed that no significant chemical changes occurred in the cellulose functional groups, indicating that the reduction in air permeability is due to the physical presence of nanoparticles rather than chemical modification of the fibers. Similar observations have been reported by other researchers who applied nanoparticles to cotton textiles [26,70,71]. However, the observed decline in air permeability in this study was only 8.8%, indicating that the treated fabric still retains adequate breathability and is likely to remain comfortable for wear.

#### 3.4.5. Tensile Strength

The impact of silver nanoparticle treatment on the mechanical properties of cotton fabric was evaluated by measuring tensile strength, as these properties are crucial for the fabric’s performance over time. The incorporation of silver nanoparticles enhanced the tensile strength while reducing the percentage elongation at break. The tensile strength increased by 32.25% from 204.01 MPa in untreated cotton fabric to 301.14 MPa in the nanoparticle-treated fabric, while elongation at break decreased by 10.47% from 8.12% to 7.27%. The increase in tensile strength can be explained by SEM observations (Figure 9b–e), which showed uniform nanoparticle coverage on the fiber surfaces that acts as a reinforcing layer, enhancing load transfer between fibers during mechanical testing. This improvement in tensile strength can be attributed to the absorption of silver nanoparticles onto the cotton fibers. In other similar studies, cotton fibers impregnated with silver nanoparticles exhibited enhanced mechanical properties due to the interaction between the nanoparticles and the hydroxyl groups of cellulose chains via hydrogen bonding or Van der Waals forces [65,72,73]. FTIR analysis (Figure 11) confirmed that the cellulose functional groups remained intact, supporting the conclusion that non-covalent interactions between nanoparticles and cellulose fibers contribute to mechanical reinforcement. Another contributing factor may be the high temperature curing process which helps secure the nanoparticles onto the fabric, further improving tensile strength. The reduction in elongation at break suggests that the addition of the nanoparticles decreases the fabric’s stretchability, making it more resistant to breaking or tearing under tension [74].

## 4. Conclusions and Recommendations

Green synthesis of silver nanoparticles was successfully performed using potato peel extracts as a reducing and stabilizing agent. The synthesized nanoparticles were predominantly spherical and effectively deposited onto 100% woven cotton fabric through an in situ approach. The process parameters—extract concentration, AgNO_3_ ratio, pH, temperature, stirring speed and reaction time—significantly influenced nanoparticles formation and uniformity. Higher extract concentrations and optimal extract to AgNO_3_ ratios enhanced the reduction of silver ions, while extreme values led to particle aggregation and polydispersity. Alkaline pH, moderate temperature (50 °C), controlled stirring (500 rpm), and a 3 h reaction time resulted in uniform nanoparticle formation with strong and well-defined SPR peaks, indicating effective and stable synthesis.

The treated fabrics exhibited strong antibacterial activity against both *S. aureus* and *E. coli*, with the effect remaining appreciable even after 20 washes. Notably, fabrics treated with silver nanoparticles outperformed those solely treated with potato peel extracts, demonstrating the enhanced efficacy of nanoparticle-based treatments. The treatment led to measurable changes in the fabric’s physical properties, including increased tensile strength and reduced air permeability, with no chemical alteration to the cellulose structure as confirmed by FTIR analysis. The observed changes in surface morphology and thermal properties further supported successful nanoparticle integration.

Overall, this work highlights the potential of using agricultural waste materials such as potato peels for the eco-friendly synthesis of functional nanomaterials. The approach offers a sustainable alternative for developing antimicrobial textiles with enhanced durability and minimal environmental impact. Future research should focus on optimizing the in situ synthesis process and enhancing nanoparticle adhesion to improve wash durability and long-term performance. Research should also focus on quantifying nanoparticle retention and leaching using ICP-MS. It is also important to evaluate antibacterial performance under real-use conditions, such as on hospital wear exposed to sweat, temperature and skin contact. Broader issues of nanoparticle leaching, potential toxicity, production cost, and regulatory approval must also be addressed to enable safe and practical application. In summary, potato-peel-mediated AgNPs show strong promise for sustainable antimicrobial textiles, but further work is needed to ensure stability, safety and feasibility in real-world applications.

## Figures and Tables

**Table 1:** Inhibitory zone of treated and untreated cotton fabrics on the tested bacteria.

Fabric Samples	Zone of Inhibition (mm) Mean ± SD	
	*E. coli*	*S. aureus*
Untreated fabric (negative control)	0.00 ± 0.00	0.00 ± 0.00
Positive control	40.00 ± 0.02	30.00 ± 0.04
PPE-treated fabric before washing	12.00 ± 0.01	8.00 ± 0.02
PPE-treated fabric after 20 washes	5.00 ± 0.00	3.50 ± 0.03
AgNP-treated fabric before washing	23.00 ± 0.05	16.00 ± 0.03
AgNP-treated fabric after 20 washes	13.00 ± 0.02	9.00 ± 0.01

## Data Availability

The original contributions presented in this study are included in the article. Further inquiries can be directed to the corresponding author (N.S.M).

## References

[1] Boryo D.E.A. (2013). The effect of microbes on textile material: A review on the way-out so far. Int. J. Eng. Sci..

[2] Sanders D., Grunden A., Dunn R.R. (2021). A review of clothing microbiology: The history of clothing and the role of microbes in textiles. Biol. Lett..

[3] Suliman Z.A., Mecha A.C., Mwasiagi J.I. (2024). Characterization and Disinfection Performance of α-Fe_2_O_3_-TiO_2_ Based Polyester Membranes for Water Treatment. Univers. J. Green. Chem..

[4] Khairy T., Amin D.H., Salama H.M., Elkholy I.M.A., Elnakib M., Gebreel H.M., Sayed H.A.E. (2024). Antibacterial activity of green synthesized copper oxide nanoparticles against multidrug-resistant bacteria. Sci. Rep..

[5] Mpofu N.S., Igadwa J.I., Nganyi E.O., Kamhala E. (2021). Antimicrobial and antiviral properties of metal nanoparticles and their potential use in textiles: A review. Ann. Univ. Oradea Fascicle Text Leatherwork.

[6] Afraz N., Uddin F., Syed U., Mahmood A. (2019). Antimicrobial Finishes for Textiles. Fash. Technol. Text. Eng..

[7] Qais F.A., Shafiq A., Khan H.M., Husain F.M., Khan R.A., Alenazi B., Alsalme A., Ahmad I. (2019). Antibacterial effect of silver nanoparticles synthesized using *Murraya koenigii* (L.) against multidrug-resistant pathogens. Bioinorg. Chem. Appl..

[8] Holubnycha V., Husak Y., Korniienko V., Bolshanina S., Tveresovska O., Myronov P., Holubnycha M., Butsyk A., Borén T., Banasiuk R. (2024). Antimicrobial Activity of Two Different Types of Silver Nanoparticles against Wide Range of Pathogenic Bacteria. Nanomaterials.

[9] Ahmed T., Ogulata R.T., Sezgin Bozok S. (2022). Silver Nanoparticles against SARS-CoV-2 and Its Potential Application in Medical Protective Clothing—A Review. J. Text. Inst..

[10] Zhao X., Li M., Zhao J., Wang X. (2024). Intelligent Sportswear Design: Innovative Applications Based on Conjugated Nanomaterials. Sci. Eng. Compos. Mater..

[11] Ibrahim N.A. (2015). Nanomaterials for Antibacterial Textiles.

[12] Hebeish A., El-Bisi M.K., El-Shafei A. (2015). Green synthesis of silver nanoparticles and their application to cotton fabrics. Int. J. Biol. Macromol..

[13] Xu J., Yıldıztekin M., Han D., Keskin C., Baran A., Baran M.F., Eftekhari A., Ava C.A., Kandemir S.İ., Cebe D.B. (2023). Biosynthesis, characterization, and investigation of antimicrobial and cytotoxic activities of silver nanoparticles using Solanum tuberosum peel aqueous extract. Heliyon.

[14] Shaheen T.I., El Aty A.A.A. (2018). In-situ green myco-synthesis of silver nanoparticles onto cotton fabrics for broad spectrum antimicrobial activity. Int. J. Biol. Macromol..

[15] Bankar A., Joshi B., Kumar A.R., Zinjarde S. (2010). Banana peel extract mediated novel route for the synthesis of silver nanoparticles. Colloids Surf. A Physicochem. Eng. Asp..

[16] Ahamed M., Majeed Khan M.A., Siddiqui M.K.J., Alsalhi M.S., Alrokayan S.A. (2011). Green synthesis, characterization and evaluation of biocompatibility of silver nanoparticles. Phys. E Low-Dimens. Syst. Nanostruct..

[17] Almadiy A.A., Nenaah G.E. (2018). Ecofriendly Synthesis of Silver Nanoparticles Using Potato Steroidal Alkaloids and Their Activity Against Phytopathogenic Fungi. Braz. Arch. Biol. Technol..

[18] Wolela A.D. (2020). Antibacterial Finishing of Cotton Textiles with Extract of Citrus Fruit Peels. Fash. Technol. Text. Eng..

[19] Ibrahim H.M.M. (2015). Green synthesis and characterization of silver nanoparticles using banana peel extract and their antimicrobial activity against representative microorganisms. J. Radiat. Res. Appl. Sci..

[20] Vigneshwaran N., Kathe A.A., Varadarajan P.V., Nachane R.P., Balasubramanya R.H. (2007). Functional finishing of cotton fabrics using silver nanoparticles. J. Nanosci. Nanotechnol..

[21] Friedman M., Huang V., Quiambao Q., Noritake S., Liu J., Kwon O., Chintalapati S., Young J., Levin C.E., Tam C. (2018). Potato Peels and Their Bioactive Glycoalkaloids and Phenolic Compounds Inhibit the Growth of Pathogenic Trichomonads. J. Agric. Food Chem..

[22] Silva-Beltrán N.P., Chaidez-Quiroz C., López-Cuevas O., Ruiz-Cruz S., López-Mata M.A., Del-Toro-sánchez C.L., Marquez-Rios E., Ornelas-Paz J.D.J. (2017). Phenolic compounds of potato peel extracts: Their antioxidant activity and protection against human enteric viruses. J. Microbiol. Biotechnol..

[23] Mpofu N.S., Mwasiagi J.I., Mecha C.A., Nganyi E.O. (2025). Evaluation of solanum tuberosum potato peel waste for use as an eco-friendly antibacterial finish for cotton fabrics. Res. J. Text. Appar..

[24] Buazar F., Bavi M., Kroushawi F., Halvani M., Khaledi-Nasab A., Hossieni S.A. (2016). Potato Extract as Reducing Agent and Stabiliser in a Facile Green One-Step Synthesis of ZnO Nanoparticles. J. Exp. Nanosci..

[25] Mahdieh Z.M., Shekarriz S., Taromi F.A. (2021). The Effect of Silver Concentration on Ag-TiO_2_ Nanoparticles Coated Polyester/Cellulose Fabric by In-situ and Ex-situ Photo-reduction Method—A Comparative Study. Fibers Polym..

[26] Čuk N., Šala M., Gorjanc M. (2021). Development of antibacterial and UV protective cotton fabrics using plant food waste and alien invasive plant extracts as reducing agents for the in-situ synthesis of silver nanoparticles. Cellulose.

[27] Harifi T., Montazer M. (2015). A review on textile sonoprocessing: A special focus on sonosynthesis of nanomaterials on textile substrates. Ultrason. Sonochem..

[28] Huang C., Cai Y., Chen X., Ke Y. (2022). Silver-based nanocomposite for fabricating high performance value-added cotton. Cellulose.

[29] Jacob P.J.S. (2022). Cotton based cellulose nanocomposites: Synthesis and application. Cotton.

[30] Patra J.K., Baek K.H. (2014). Green Nanobiotechnology: Factors Affecting Synthesis and Characterization Techniques. J. Nanomater..

[31] Seifipour R., Nozari M., Pishkar L. (2020). Green Synthesis of Silver Nanoparticles using Tragopogon Collinus Leaf Extract and Study of Their Antibacterial Effects. J. Inorg. Organomet. Polym. Mater..

[32] Ashraf J.M., Ansari M.A., Khan H.M., Alzohairy M.A., Choi I. (2016). Green synthesis of silver nanoparticles and characterization of their inhibitory effects on AGEs formation using biophysical techniques. Sci. Rep..

[33] Kredy M.H. (2018). The effect of pH, temperature on the green synthesis and biochemical activities of silver nanoparticles from Lawsonia inermis extract. J. Pharm. Sci. Res..

[34] Aladpoosh R., Montazer M., Samadi N. (2014). In situ green synthesis of silver nanoparticles on cotton fabric using *Seidlitzia rosmarinus* ashes. Cellulose.

[35] Liu H., Lee Y.Y., Norsten T.B., Chong K. (2014). In situ formation of anti-bacterial silver nanoparticles on cotton textiles. J. Ind. Text..

[36] (2006). Textiles—Tests for Colour Fastness—Part c10: Colour Fastness to Washing with Soap or Soap and Soda.

[37] Jain A., Kongkham B., Puttaswamy H., Butola B.S., Malik H.K., Malik A. (2022). Development of Wash-Durable Antimicrobial Cotton Fabrics by In Situ Green Synthesis of Silver Nanoparticles and Investigation of Their Antimicrobial Efficacy against Drug-Resistant Bacteria. Antibiotics.

[38] (1995). Textiles—Determination of the Permeability of Fabrics to Air.

[39] (2013). Textiles—Tensile properties of fabrics—Part 1: Determination of maximum force and elongation at maximum force using the strip method.

[40] Ahmed S., Saifullah, Ahmad M., Swami B.L., Ikram S. (2016). Green synthesis of silver nanoparticles using Azadirachta indica aqueous leaf extract. J. Radiat. Res. Appl. Sci..

[41] De Leersnyder I., Rijckaert H., De Gelder L., Van Driessche I., Vermeir P. (2020). High variability in silver particle characteristics, silver concentrations, and production batches of commercially available products indicates the need for a more rigorous approach. Nanomaterials.

[42] Djuhana D., Putra M.H., Imawan C., Fauzia V., Harmoko A., Handayani W., Ardani H. (2016). Numerical study of the plasmonic resonance sensitivity silver nanoparticles coated polyvinyl alcohol (PVA) using Bohren-Huffman-Mie (BHMie) approximation. AIP Conf. Proc..

[43] Verma A., Mehata M.S. (2016). Controllable synthesis of silver nanoparticles using Neem leaves and their antimicrobial activity. J. Radiat. Res. Appl. Sci..

[44] Tyavambiza C., Elbagory A.M., Madiehe A.M., Meyer M., Meyer S. (2021). The antimicrobial and anti-inflammatory effects of silver nanoparticles synthesised from cotyledon orbiculata aqueous extract. Nanomaterials.

[45] Kaur R., Avti P., Kumar V., Kumar R. (2021). Effect of various synthesis parameters on the stability of size controlled green synthesis of silver nanoparticles. Nano Express.

[46] Melkamu W.W., Bitew L.T. (2021). Green synthesis of silver nanoparticles using *Hagenia abyssinica* (Bruce) J.F. Gmel plant leaf extract and their antibacterial and anti-oxidant activities. Heliyon.

[47] Azarbani F., Shiravand S. (2020). Green synthesis of silver nanoparticles by *Ferulago macrocarpa* flowers extract and their antibacterial, antifungal and toxic effects. Green Chem. Lett. Rev..

[48] Mahiuddin M., Saha P., Ochiai B. (2020). Green synthesis and catalytic activity of silver nanoparticles based on piper chaba stem extracts. Nanomaterials.

[49] Rao B., Tang R.C. (2017). Green synthesis of silver nanoparticles with antibacterial activities using aqueous *Eriobotrya japonica* leaf extract. Adv. Nat. Sci. Nanosci. Nanotechnol..

[50] Jang M.H., Lee S., Hwang Y.S. (2015). Characterization of silver nanoparticles under environmentally relevant conditions using asymmetrical flow field-flow fractionation (AF4). PLoS ONE.

[51] Elamawi R.M., Al-Harbi R.E., Hendi A.A. (2018). Biosynthesis and characterization of silver nanoparticles using *Trichoderma longibrachiatum* and their effect on phytopathogenic fungi. Egypt. J. Biol. Pest. Control..

[52] Jalab J., Abdelwahed W., Kitaz A., Al-Kayali R. (2021). Green synthesis of silver nanoparticles using aqueous extract of *Acacia cyanophylla* and its antibacterial activity. Heliyon.

[53] Erdogan O., Abbak M., Demirbolat G.M., Birtekocak F., Aksel M., Pasa S., Cevik O. (2019). Green synthesis of silver nanoparticles via *Cynara scolymus* leaf extracts: The characterization, anticancer potential with photodynamic therapy in MCF7 cells. PLoS ONE.

[54] Samimi S., Maghsoudnia N., Eftekhari R.B., Dorkoosh F. (2018). Lipid-Based Nanoparticles for Drug Delivery Systems.

[55] Jagtap U.B., Bapat V.A. (2013). Green synthesis of silver nanoparticles using *Artocarpus heterophyllus* Lam. seed extract and its antibacterial activity. Ind. Crop. Prod..

[56] Balamurugan M., Saravanan S., Soga T. (2017). Coating of green-synthesized silver nanoparticles on cotton fabric. J. Coat. Technol. Res..

[57] Zhou Q., Chen J., Lu Z., Tian Q., Shao J. (2022). In Situ Synthesis of Silver Nanoparticles on Flame-Retardant Cotton Textiles Treated with Biological Phytic Acid and Antibacterial Activity. Materials.

[58] Mahmud S., Pervez N., Taher M.A., Mohiuddin K., Liu H.H. (2020). Multifunctional organic cotton fabric based on silver nanoparticles green synthesized from sodium alginate. Text. Res. J..

[59] Shateri-Khalilabad M., Yazdanshenas M.E., Etemadifar A. (2017). Fabricating multifunctional silver nanoparticles-coated cotton fabric. Arab. J. Chem..

[60] Syafiuddin A., Fulazzaky M.A., Salmiati S., Roestamy M. (2020). Sticky silver nanoparticles and surface coatings of different textile fabrics stabilised by *Muntingia calabura* leaf extract. SN Appl. Sci..

[61] Novoa C.C., Tortella G., Seabra A.B., Diez M.C., Rubilar O. (2022). Cotton Textile with Antimicrobial Activity and Enhanced Durability Produced by L -Cysteine-Capped Silver nanoparticles. Processes.

[62] Tania I.S., Ali M., Bhuiyan R.H. (2020). Experimental Study on Dyeing Performance and Antibacterial Activity of Silver Nanoparticle-Immobilized Cotton Woven Fabric. Autex Res. J..

[63] Salama A., Abouzeid R.E., Owda M.E., Cruz-Maya I., Guarino V. (2021). Cellulose–silver composites materials: Preparation and applications. Biomolecules.

[64] Krishnamoorthy K., Navaneethaiyer U., Mohan R., Lee J., Kim S.J. (2012). Graphene oxide nanostructures modified multifunctional cotton fabrics. Appl. Nanosci..

[65] Ibrahim H.M.M., Hassan M.S. (2016). Characterization and antimicrobial properties of cotton fabric loaded with green synthesized silver nanoparticles. Carbohydr. Polym..

[66] Jagadeshvaran P.L., Bose S. (2023). Nano silver-deposited cotton textile core with carbon nanostructure-filled shell for suppression of electromagnetic radiation via absorption-reflection-absorption. Mater. Chem. Phys..

[67] Nam S., Baek I.S., Hillyer M.B., He Z., Barnaby J.Y., Condon B.D., Kim M.S. (2022). Thermosensitive textiles made from silver nanoparticle-filled brown cotton fibers. Nanoscale Adv..

[68] Ogulata R.T. (2006). Air Permeability of Woven Fabrics. J. Text. Appar. Technol. Manag..

[69] Onal L., Yildirim M. (2012). Comfort properties of functional three-dimensional knitted spacer fabrics for home-textile applications. Text. Res. J..

[70] Ali A., Nguyen N.H.A., Baheti V., Ashraf M., Militky J., Mansoor T., Noman M.T., Ahmad S. (2018). Electrical conductivity and physiological comfort of silver coated cotton fabrics. J. Text. Inst..

[71] Noman M.T., Petru M., Amor N., Louda P. (2020). Thermophysiological comfort of zinc oxide nanoparticles coated woven fabrics. Sci. Rep..

[72] Gollapudi V.R., Mallavarapu U., Seetha J., Akepogu P., Amara V.R., Natarajan H., Anumakonda V. (2020). In situ generation of silver and silver oxide nanoparticles on cotton fabrics using *Tinospora cordifolia* as bio reductant. SN Appl. Sci..

[73] Xu Q.B., Xie L.J., Diao H., Li F., Zhang Y.Y., Fu F.Y., Liu X.D. (2017). Antibacterial cotton fabric with enhanced durability prepared using silver nanoparticles and carboxymethyl chitosan. Carbohydr. Polym..

[74] Elmogahzy Y., Farag R. (2018). Tensile properties of cotton fibers: Importance, research, and limitations. Handbook of Properties of Textile and Technical Fibres.

